# Supplementary material for: Identification of Neoadjuvant Chemotherapy Response in Muscle-Invasive Bladder Cancer by Fourier-Transform Infrared Micro-Imaging
Source: Cancers (Basel). 2021 Dec 21;14(1):21. doi: 10.3390/cancers14010021 (PMC8750189; doi:10.3390/cancers14010021)
Supplement: Supplementary file 1 [file cancers-14-00021-s001.zip › cancers-1474327-supplementary.pdf]

cancers MDPI

Supplementary Materials

Identification of neoadjuvant chemotherapy response in muscle-invasive bladder cancer by Fourier-Transform infrared micro-imaging

Camille Mazza, Vincent Gaydou, Jean-Christophe Eymard, Philippe Birembaut, Valérie Untereiner, Jean-François Côté, Isabelle Brocheriou, David Coeffic, Philippe Villena, Stéphane Larré, Vincent Vuiblet and Olivier Piot

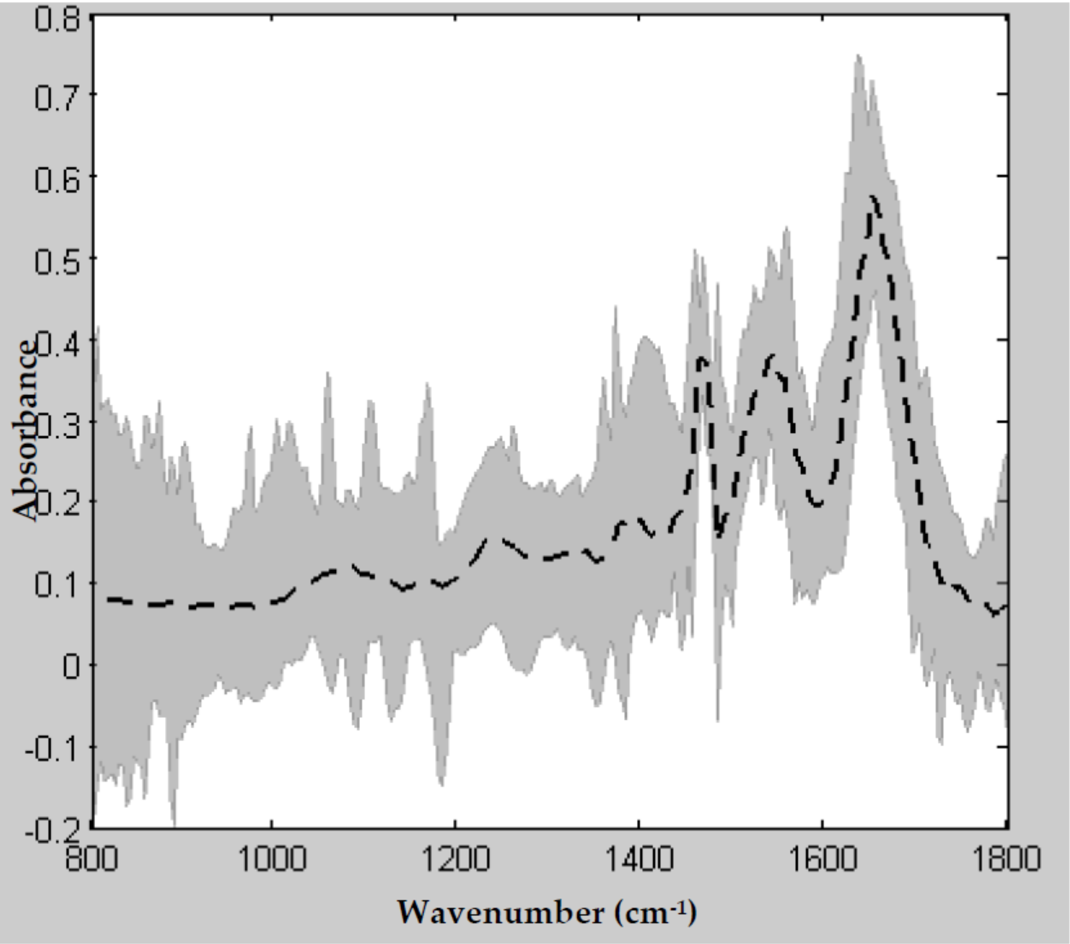

Figure S1. Whole dataset after the pre-processing step that includes reduction on the 800–1800 cm^{-1} spectral range and EMSC. The spectrum in the dotted line corresponds to the target spectrum (exhibiting vibrations associated with the protein content such as Amide I and Amide II bands but also paraffin signal around 1450 cm^{-1}) and the grey area the maximal variations of intensity observed at each wavenumber. EMSC aims at neutralizing the variability of the paraffin signal, eliminating outlier spectra, and normalizing the data according to the target spectrum.

Copyright: © 2021 by the authors. Licensee MDPI, Basel, Switzerland. This article is an open access article distributed under the terms and conditions of the Creative Commons Attribution (CC BY) license (http://creativecommons.org/licenses/by/4.0/).
